# An assessment of factors controlling N_2_O and CO_2_ emissions from crop residues using different measurement approaches

**DOI:** 10.1007/s00374-017-1195-z

**Published:** 2017-04-13

**Authors:** Giuseppe Badagliacca, Paolo Ruisi, Robert M. Rees, Sergio Saia

**Affiliations:** 1 Dipartimento di Scienze Agrarie e Forestali, Università degli Studi di Palermo, Viale delle Scienze, 90128 Palermo, Italy; 2 Scotland’s Rural College (SRUC), West Mains Road, Edinburgh, EH9 3JG UK; 3 Council for Agricultural Research an Economics (CREA)–Cereal Research Centre (CREA-CER), S.S. 673, km 25,200, 71122 Foggia, Italy

**Keywords:** Crop residues, Carbon dioxide, Greenhouse gas, Nitrous oxide, Residue decomposition

## Abstract

Management of plant residues plays an important role in maintaining soil quality and nutrient availability for plants and microbes. However, there is considerable uncertainty regarding the factors controlling residue decomposition and their effects on greenhouse gas (GHG) emissions from the soil. This uncertainty is created both by the complexity of the processes involved and limitations in the methodologies commonly used to quantify GHG emissions. We therefore investigated the addition of two soil residues (durum wheat and faba bean) with similar C/N ratios but contrasting fibres, lignin and cellulose contents on nutrient dynamics and GHG emission from two contrasting soils: a low-soil organic carbon (SOC), high pH clay soil (Chromic Haploxerert) and a high-SOC, low pH sandy-loam soil (Eutric Cambisol). In addition, we compared the effectiveness of the use of an infrared gas analyser (IRGA) and a photoacoustic gas analyser (PGA) to measure GHG emissions with more conventional gas chromatography (GC). There was a strong correlation between the different measurement techniques which strengthens the case for the use of continuous measurement approaches involving IRGA and PGA analyses in studies of this type. The unamended Cambisol released 286% more CO_2_ and 30% more N_2_O than the Haploxerert. Addition of plant residues increased CO_2_ emissions more in the Haploxerert than Cambisol and N_2_O emission more in the Cambisol than in the Haploxerert. This may have been a consequence of the high N stabilization efficiency of the Haploxerert resulting from its high pH and the effect of the clay on mineralization of native organic matter. These results have implication management of plant residues in different soil types.

## Introduction

Agriculture forestry and related land uses are responsible for just under 25% of global greenhouse gas emissions (IPCC 2014). Agronomic practices are recognized as key opportunities to reduce greenhouse gas (GHG) emissions (particularly for N_2_O and CO_2_). The addition of plant residues to the soil from crops and cover crops is of significant importance to crop management strategies to enhance soil organic C (SOC) and soil fertility and to offset agricultural GHG emissions (Lugato et al. 2014). However, decomposition of residues will also add nitrogen (N) to the soil, and with the default N_2_O emission factor of 1% of the added N in the IPCC (2006) methodology, a proportional increase in estimated N_2_O emissions is predicted. However, IPCC predictions have been developed around limited experimental data and recent studies indicate that default emission factors may overestimate residue N_2_O emissions (Jeuffroy et al. 2013). GHG emission after crop residue amendment is related to both its decomposition and the microbial activity of soil and depends from several factors linked to the environment, soil proprieties and crop residue traits (Aulakh et al. 1991; Powlson et al. 2011). In particular, the GHG emission from the soil is mediated by soil porosity (Killham et al. 1993), pH (Mørkved et al. 2006), organic C and N content (Hayakawa et al. 2009), microbial community (Graf et al. 2016), texture (Chen et al. 2013), soil temperature (Kesik et al. 2006) and moisture content, all of which regulate gas production processes and emission (Skiba and Ball 2002; Rees et al. 2013). Moreover, crop residue addition to the soil can also indirectly affect GHG emissions, providing a source of readily available C and N in the soil, stimulating microbial activity (Aulakh et al. 2001; Huang et al. 2004), promoting the decomposition of native SOC and altering soil aeration, water holding capacity, oxidation and denitrification processes in the soil (Fontaine et al. 2004; Derrien et al. 2014).

With regard to the crop residue characteristics, the most important property is its C/N ratio that determines organic N dynamics in the soil (Heal et al. 1997; Baggs et al. 2003; Al-Kaisi and Yin 2005; Garcia-Ruiz and Baggs 2007). In particular, it has been shown that higher N_2_O emissions occur from soil after the incorporation of residues with low C/N ratio, such as legumes, rather than after cereal straw as a result of mineralization processes (Baggs et al. 2000; Huang et al. 2004; Raiesi 2006). On the contrary, low N_2_O emissions were reported following the application of crop residues with high C/N ratios (Gentile et al. 2008). However, it has also been shown that the incorporation of crop residues with high C/N ratios may provide the energy for the denitrification process, and this can increase N_2_O emissions (Sarkodie-Addo et al. 2003). Other crop residue properties can play an important role on the decomposition process influencing microbial activity, including lignin (Palm and Rowland 1997), lignin/N ratios (Curtin et al. 1998) polyphenol (Muhammad et al. 2010), water-soluble phenolic contents (Palm and Rowland 1997), percentages of soluble C and N (Cogle et al. 1989) and neutral detergent fibre (NDF). This understanding of the multiple drivers responsible for GHG emissions from crop residues is helpful in interpreting research findings. Baggs et al. (2000) found an increase of N_2_O emissions after lettuce incorporation into the soil due to its low C/N ratio. However, Tanveer et al. (2014) and Zou et al. (2004) observed a reduction of N_2_O emission after incorporation of low C/N crop residue of corn and rice straw. This apparent contradiction may be a consequence of the interaction of multiple factors controlling emission. Shan and Yan (2013), in a meta-analysis, reported that the application of canola, bean and lettuce residues increased N_2_O emissions more than with other kinds of crop residues. With regard to CO_2_ emissions, Muhammad et al. (2010) observed higher emissions in soil amended with alfalfa than with sugarcane, maize, sorghum and cotton and attributed such result to a release of more easily degradable and soluble C in alfalfa than with other crop residues. Chen et al. (2015) observed a general increase of CO_2_ emissions from a soil amended with different types of residues but with higher cumulative emissions in peanuts, soybean and maize than in other cereals due to their higher N and lower NDF content.

An accurate quantification of CO_2_ and N_2_O emission following return of crop residues to soils is required to develop efficient strategies to reduce the environmental impact of farming practices. Presently, static chamber methods coupled with gas chromatography (GC) analysis are the most widely technique used to quantify N_2_O losses in field and laboratory experiments. However, the method is time consuming and entails a wide series of operations from the manual sampling to the laboratory analysis, introducing errors and making difficult to implement high-resolution monitoring over time (Tirol-Padre et al. 2014). In order to obtain high-resolution temporal data, infrared gas analyser (IRGA) and photoacoustic gas analyser (PGA) have been used in agricultural GHG emission studies (Luo and Zhou 2006; Lawrence et al. 2009; Stackhouse et al. 2011). IRGA allows to measure CO_2_ fluxes using an infrared sensor, and PGA is a photoacoustic infrared multi-gas monitoring system that allows to measure simultaneously CO_2_, N_2_O and CH_4_. Measurement of CO_2_ efflux by IRGA systems is usually based on different methodologies proposed by the manufacturing companies, and there is not an internationally recognized protocol creating uncertainties in the comparison between different instruments (Mills et al. 2011). PGA has been widely used in field experiments, and several authors found a high correlation between CO_2_ and N_2_O measurements made with PGA and GC (De Klein et al. 2008; Iqbal et al. 2013). Other authors reported an overestimation of emission on the data obtained with PGA than GC (Yamulki and Jarvis 1999). Furthermore, the precision of measurement may also depend from the soil type and soil cover, which can affect the assessment of emission spatial variability. The precision of the various instruments (IRGA and PGA compared to the widely used CG) in measuring GHG emission has never been measured. However, in contrast to the GC-based methodology, these systems are able to provide a continuous measurement of the GHG emission, thus allowing to better study the trend of the emission from the soil and its relationship with agronomical management techniques and environmental variability. In addition, IRGA and PGA have not been previously directly compared.

Soil GHG emissions from Cambisols which occur widely in cool temperate climates have widely studied in the past whereas the effect of soil characteristics typical of the Mediterranean such as Vertisols, with their high clay content high pH and low organic carbon content, on crop residue decomposition and gaseous emissions is less known. The aims of the present study were (i) to evaluate the short-term emissions of N_2_O and CO_2_ after the addiction of two crop residues with different structural fibre compositions (either faba bean and wheat), in two soils with contrasting proprieties, a Chromic Haploxerert with a high clay content and a Eutric Cambisol with a sandy-loam texture and (ii) assess the flexibility of two systems for the high temporal resolution measurements (IRGA and PGA), to measure soil GHG emissions from soils with different emission levels in controlled conditions. Experiments were undertaken in a controlled pot setup over a short period and in the absence of plants in order to simulate the effects of crop residues between cropping cycles. These conditions avoided strong time-related variation in the emission due to the impoverishment of the ready available N pool and living plant C inputs to and mineral uptake from soil, which could have altered the emission rates.

## Materials and methods

An experiment was established during 2014 in controlled environment conditions at Scotland’s Rural College (SRUC) Edinburgh. A complete randomized factorial design with three replicates was adopted. Treatments were soil Eutric Cambisol and Chromic Haploxerert (Vertisol) and the kinds of plant residue added were as follows: faba bean residue, durum wheat residue or unamended control. The Cambisol was collected at nine locations per plot from the top 20 cm at Bush Estate (lat, 55° 51′ N, long, 3° 12′ W; 199 m a.s.l.) near Edinburgh (Scotland); the Haploxerert (Vertisol) was collected at the Pietranera farm (37° 30′ N, 13° 31 E; 178 m a.s.l.) in Santo Stefano Quisquina (Sicily). Both soils were sampled in early October 2014. Soil was collected from conventional tilled experimental plots at the Bush Estate in Scotland and from conventionally tilled plots at Pietranera farm in Sicily (Table 1). At both sites, the soil was collected in plots previously cultivated with cereals (wheat in Sicily and barley in Scotland). Further information regarding the soil sampling sites is available in Vinten et al. (1992) and Amato et al. (2013), respectively. Before establishing the experiment, soil was air-dried and passed through a 2-mm mesh and visible roots and organic residues were removed and then mixed thoroughly before use; water holding capacity of both soils was measured on a weight basis. Oven-dried crop biomass of wheat (cv. Simeto) and faba bean (cv. Gemini) (see Table 2 residues traits), cultivated at Pietranera farm, was ground to pass a 1-mm screen, mixed and used as crop residues.

**Table 1:** Main properties of soils

Soil properties	Scotland bush estate	Sicily Pietranera
Soil classification	Eutric Cambisol	Chromic Haploxerert (Vertisol)
Soil series	Macmerry	Gessoso-Solfifera (sulphurous-chalky)
Texture	Sandy-loam	Clay-loam
Coordinates	55.9 N, 3.2 W	37.3 N, 13.3 W
Altitude	199	178
Slope (%)	6	7
Clay (%)	12.7	52.5
Silt (%)	15.7	21.6
Sand (%)	71.6	25.9
pH	6.6	8.1
Field capacity (pF 2.5) (%)	36	38
Permanent wilting point (pF 4.5) (%)	20	16
Organic matter (%)	4.3	2.4
Total N (%)	0.21	0.13

**Table 2:** Composition of crop residues

Chemical properties of crop residues	Faba bean	Durum wheat
Organic matter	91.8	92.1
N content	1.4	1.3
Crude protein	8.8	8.1
Ether extract	1.1	1.7
Acid detergent fibre (ADF)	48.0	28.8
Acid detergent lignin (ADL)	10.0	3.5
Cellulose	38	25.3
Neutral detergent fibre (NDF)	54.0	45.4
Hemicellulose	6	16.6
Ash	8.2	7.9
ADL ash	0.4	3.2

Pots were 10 cm in diameter and 25-cm height and were filled with 1.5 kg of soil to achieve a bulk density of 1.25 g cm^−3^. Crop residues were mixed with the soil at a rate of 5 g crop residue per kilogram of soil. The bottom part of the pot (15–25-cm depth) was filled with sand. Then, pots were brought to 60–70% of the water holding capacity. After each sampling, an amount of water corresponding to the evaporation losses was added to each pot and the pots were randomized inside the greenhouse. During the experiment, soil temperature was recorded using a temperature data logger (EL-USB-3, Lascar Electronics, UK).

Both CO_2_ and N_2_O soil emissions were measured three times per week, on 22 sampling occasions, by means of two different methods: an online infrared gas analyser (IRGA, EGM-4 CO_2_, PP system, USA) and a photoacoustic gas analyser (PGA, INNOVA 1412, LumaSense Technologies A/S, USA). Measurements were always taken between the 9:00 and the 15:00, and each time, the equipment order was reversed. The IRGA was equipped with a SRC-1 soil respiration chamber equipped with a fan, with of 10 cm of diameter and 15-cm height, sealed on top of the pot by an airtight rubber. The air from the chamber was send to the analyser at a flow rate of 0.1 l min^−1^. After 15 s of flushing, the chamber was placed above the pot, equilibrated for 15 s, then the CO_2_ concentration was measured every 5 s and the flux was calculated from the concentration increase over time until a good linear fit was obtained.

The PGA was equipped with a PVC chamber with 10 cm of diameter and 10 cm height, connected to the equipment by two small rubber pipes on the chamber top, and sealed above the pot by a rubber seal. The analyser automatically pumped ∼0.1 l min^−1^ of air from inside the chambers and performed the analysis with a 5-s sampling integration time and a fixed flushing time of 8 s for the chamber and 3 s for the tubing. The PGA instrument was calibrated in the lab for CO_2_ and N_2_O by the LumaSense technologies company, with a gas concentration of 3496.8 ppm for CO_2_ and 51.32 ppm for N_2_O, and its detection limits were 1.5 ppm for CO_2_ and 0.03 ppm for N_2_O. The equipment performed a built-in compensation for water and cross interferences. Before the flux measurements, the instrument analysed ambient air for about 30 min until readings for CO_2_ and N_2_O were stable. The overall time for sampling and measurement of CO_2_ and N_2_O concentration and dew-point temperature was approximately 70 s; each measurement was made every 2 min.

Gas flux measurement (CO_2_ from both IRGA and PGA and N_2_O from PGA), in two different periods during the experiment, was compared with analyses by GC in order to confirm the reliability of the instruments. CO_2_ and N_2_O emissions were measured using the static closed chamber technique (Hutchinson and Mosier 1981). A chamber of polyvinyl chloride (PVC) with 10 cm of diameter and 15-cm height and a lid with a gas sampling port was sealed above each pot for 60 min. Before and after this period, gas samples were collected in portable evacuated glass vials (Chadwick et al. 2014), transported to the lab and analysed by a GC (Agilent 7890a, Agilent Technologies Ltd., Stockport, UK) equipped with a thermal conductivity detector (TCD, detection limit for CO_2_ of 23.9 ppm) and an electron capture detector (ECD, detection limit for N_2_O of 0.074 ppm). Fluxes of CO_2_ and N_2_O were calculated from the increase in concentration in the chamber corrected for the chamber air temperature using the following relation (Jantalia et al. 2008):

$$ f=\frac{\varDelta C}{\varDelta t}\times \frac{V}{A}\times \frac{m}{ V m} $$

where ∆*C*/∆*t* is the gas increment during the chamber closure time, *V* is the volume of the chamber, *A* is the soil area, *m* is the molecular weight of the gases, and *Vm* is the gas molar volume corrected for the ambient temperature.

The total amounts of N_2_O and CO_2_ emissions were calculated by linear interpolation between consecutive using the following equation (Cai et al. 2012):

$$ \mathrm{Cumulative}\ \mathrm{emission}\ \mathrm{of}\ {\mathrm{N}}_2\mathrm{O}\ \mathrm{or}\ {\mathrm{CO}}_2=\sum_{i=1}^n\left({F}_i+{F}_{i+1}\right)/2\times \left({t}_{i+1}-{t}_i\right)\times 24 $$

where *F* is the emission flow of N_2_O and CO_2_ at the *i* ^th^ measurement, (*t*_*i* + 1_ − *t*_*i*_) is the time length between two adjacent measurements, and *n* is the total measurement number.

Plant dry matter (oven drying), ether extract (Method 920.39, diethyl ether, traditional Soxhlet extraction), total N (Kjeldahl) and crude protein (calculated from the total N by standard Jones factor, *N* × 6.25) were analysed following methods described by AOAC (1995). NDF, acid detergent fibre (ADF), acid detergent lignin (ADL), cellulose and hemicellulose were analysed following the sequential method proposed by Van Soest et al. (1991) and using a Fibertec System M 1020 extractor (Foss, Höganäs). The soluble fraction was obtained by boiling 1 g of ground residues in deionized water (100 °C) for 30 min followed by extraction with a neutral detergent (EDTA and Na lauryl sulphate at 100 °C) for 60 min to obtain the NDF fraction. ADF extraction was performed by boiling the sample for 60 min in an acid detergent solution (cetyltrimethylammonium (CTAB) in H_2_SO_4_). Then, the residual detergent was removed by washing the sample with hot water. Finally, the ADF was then treated with 72% H2SO4 (*w*/*w*) for 3 h at ambient temperature and the final mass of the non-extractable fraction was considered as lignin (ADL). Cellulose was calculated as the difference between ADF and ADL while hemicellulose as the difference between NDF and ADF. Ash and ADL ash measurements were performed at 550 °C for 4 h. For each residue type, the analyses were performed in triplicate. Total C of biomasses and soils was analysed by an automated analyser (Flash 2000, Thermo Finnigan, Glasgow, UK).

At the end of the experiment, two soil samples from each pot were collected: one from the top to 5-cm depth and the other from 5- to 15-cm depth. Soil pH was measured in a 1:5 (*v*/*v*) suspension of soil in water. Dissolved organic C (DOC) content in the soil was determined by a total organic C analyser (DC-80, Rosemount Analytical, Inc. Dohrmann Division, USA) after the removal of inorganic C by acidifying the sample. Concentrations of NH_4_^+^-N and NO_3_^−^-N were determined from 10 g of soil extracted with 100 ml of 2 M KCl (1:5 ratio); then, the filtered extract NH_4_^+^-N and NO_3_^−^-N concentrations were measured by a continuous flow analysis autoanalyser (SAN SYSTEM, Skalar Analytical B.V., Netherlands).

Analysis of variance (ANOVA) was undertaken using a mixed model according to the statistical design in SAS environment (SAS Institute 2008). Treatment means were separated using *p* differences of the LSMEANS.

Regressions between GC and IRGA, and GC and PGA, for CO_2_, and for CO_2_ and N_2_O, respectively, were computed. Soil CO_2_ emission rate measurements from IRGA and PGA were compared on the 22 sampling occasions. Comparisons were made by a regression analysis and the index of agreement (IoAd) (SAS Institute 2008; Bennett et al. 2013).

## Results

**Fig. 1:**
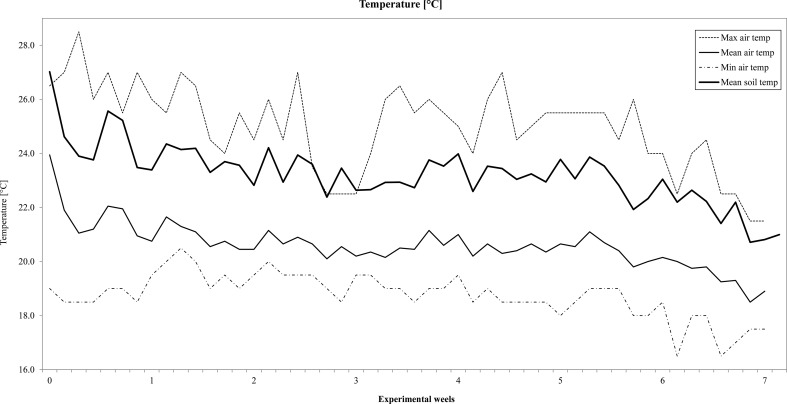
Daily minimum, maximum, and mean air temperatures in the greenhouse and mean soil temperature during the experiment

The temperature inside the greenhouse during the experiment ranged from a minimum of 17 °C to the maximum of 28.5 °C, with an average of 20.5 °C, while soil temperature ranged from a maximum of 27 °C to a minimum of 20 °C with a slight decreasing trend from the start to the end of the experiment (Fig. 1). The chemical composition of the plant residues used in the present study, expressed as percentages, are reported in Table 2. The N contents of faba bean and durum wheat were comparable (1.4 vs 1.3%, respectively). With regard to the other constituents, marked differences were found between the plant residues. In particular, faba bean had higher ADF (+66%), ADL (+186%), cellulose (+60%) and NDF (+19%) than wheat and a lower content of hemicellulose (−51%) (Table 2).

### Carbon and nitrogen dynamics

The Haploxerert used in the present study had a high pH (8.1) and high clay and low total C content (1.39%), whereas the Cambisol had a near neutral (6.6), low clay and high C content (2.48%). Interaction between soil and residue type for these soil properties by the end of the experiment was strong and significant (*p* < 0.05) (Table 3). As expected, the addition of organic residues mostly increased DOC in both the top and sub-soil layers of the Haploxerert (on average by 52.5% compared to unamended control), whereas there was no significant effect on the Cambisol.

**Table 3:** Effect of the addition of two crop residues (durum wheat or faba bean, and unamended control) on dissolved organic C (DOC), pH, NH_4_^+^-N and NO_3_^−^-N content and NH_4_^+^-N/ NO_3_^−^-N ratios in 0–5- and 5–15-cm soil layers of Chromic Haploxerert and Eutric Cambisol soils

		Chromic Haploxerert			Eutric Cambisol			*p* value		
		Faba bean	Durum wheat	No addition	Faba bean	Durum wheat	No addition	Soil	Residue type	*S × T*
0–5-cm soil layer										
DOC	mg C kg^−1^ soil	42.5	43.2	33.6	73.5	67.6	67.2	<0.001	0.000	0.007
pH	−	7.7	7.8	7.8	5.8	5.4	5.9	<0.001	0.009	0.019
NH_4_^+^-N	mg N kg^−1^ soil	1.6	3.3	1.7	0.9	1.3	0.9	<0.001	<0.001	<0.001
NO_3_^−^-N	mg N kg^−1^ soil	0.4	2.4	0.3	104.6	149.6	164.5	<0.001	0.001	0.001
NH_4_^+^-N/NO_3_^−^-N	−	4.3	1.4	6.5	0.009	0.008	0.006	<0.001	0.001	0.001
5–15-cm soil layer										
DOC	mg C kg^−1^ soil	75.9	83.0	48.1	86.4	93.1	91.8	<0.001	<0.001	<0.001
pH	−	7.7	7.7	7.8	5.9	5.6	5.8	<0.001	0.037	0.043
NH_4_^+^-N	mg N kg^−1^ soil	13.5	32.0	5.8	1.1	1.5	0.9	<0.001	<0.001	<0.001
NO_3_^−^-N	mg N kg^−1^ soil	0.5	0.5	0.8	36.9	43.3	66.4	<0.001	<0.001	<0.001
NH_4_^+^-N/NO_3_^−^-N	−	25.7	62.9	7.3	0.030	0.034	0.014	<0.001	<0.001	<0.001

The soil incubation, either with or without plant residue incorporation, decreased soil pH by 0.86 in the Cambisol and 0.33 in the Haploxerert. The effect of the addition of organic residues to the soil pH varied with both the soil and kind of biomass incorporated: in the Cambisol, addition of wheat residues significantly decreased pH in the top and sub-layers when compared with the unamended control whereas addition of faba bean residues did not influence soil pH. In the Haploxerert, no effects of the addition of organic residues on soil pH were found in both soil layers.

The concentration of NH_4_^+^-N was higher in the Haploxerert than Cambisol, and this is particularly apparent in the sub-layer. The role of the addition of organic residues on soil NH_4_^+^-N depended on the soil and kind of biomass added: addition of durum wheat residues increased soil ammonium-N in top layer of both soils (+40% in the Cambisol and +102% in the Haploxerert), whereas NH_4_^+^-N in the soils amended with faba bean residues was similar to those of the controls. In the sub-layer of the Cambisol, the effect of the addition of the organic residues was similar to that observed in the top layer, whereas the addition of both residues strongly increased the NH_4_^+^-N of Haploxerert compared to the unamended control (+133% in faba bean and +454% in wheat residues).

The concentration of NO_3_^−^-N in both layers was significantly higher in the Cambisol when compared with the Haploxerert, and this occurred irrespective of the addition of organic residues. In the Cambisol, addition of faba bean residues reduced NO_3_^−^-N more than wheat residues, especially in the sub-layer, when compared with the unamended control. In the Haploxerert, NO_3_^−^-N in both layers did not vary with the addition of plant residues.

NH_4_^+^-N/NO_3_^−^-N ratio differed considerably in the different soil types: in the unamended controls, it was 6.467 in the Haploxerert and 0.006 in the Cambisol. In the latter, addition of organic residues to the soil did not influence the NH_4_^+^-N/NO_3_^−^-N of either the top or sub-layer. In the top layer of Haploxerert, the addition of organic residues reduced the NH_4_^+^-N/NO_3_^−^-N ratio, especially when faba bean residues were added. In the sub-layer, an opposite result was found, and thus, addition of organic residues increased the NH_4_^+^-N/NO_3_^−^-N ratio, especially when wheat residues were added.

### Greenhouse gas emissions

**Fig. 2:**
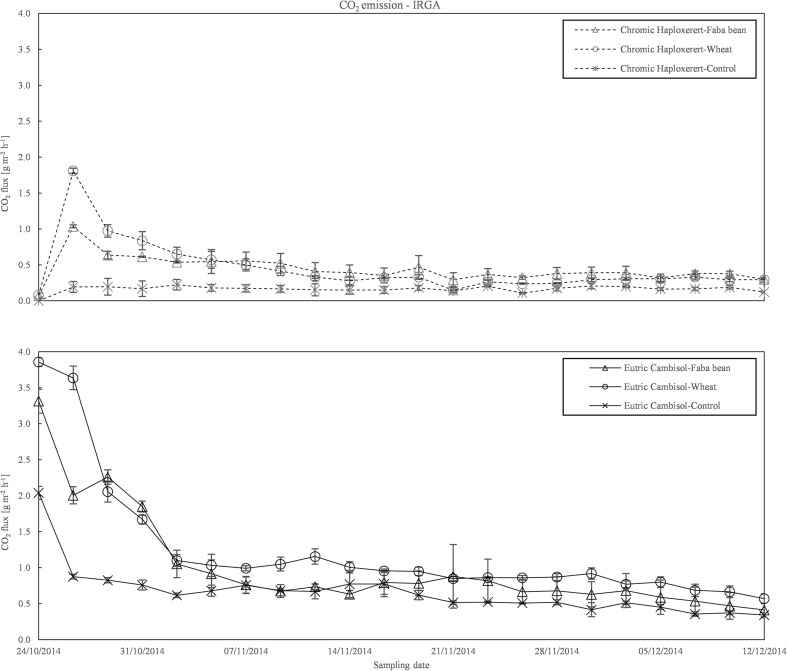
CO_2_ emission course from Chromic Haploxerert and Eutric Cambisol amended with faba bean and wheat residues, or unamended (control), measured with IRGA during the experimental period. Data are means ± SE (*n* = 3)

Carbon dioxide fluxes, measured with IRGA, ranged from a minimum value of 0.11 g m^−2^ h^−1^ to a maximum value of 3.64 g m^−2^ h^−1^ (Fig. 2). For almost the entire experimental period, the Cambisol had a higher CO_2_ emission flux than the Haploxerert. At the beginning of the experiment, the two soils reached the maximum emission flux at the first and second days of measurement with fluxes of 3.58 g m^−2^ h^−1^ for the Cambisol and 1.42 g m^−2^ h^−1^ for the Haploxerert.

The highest CO_2_ fluxes were recorded in both soils amended with wheat straw whereas the lowest in the unamended controls. The differences in emission between the two soils were strong in the first 2 weeks of measurement, where the 53.8 and 46.2% of total CO_2_ were emitted from the Cambisol and the Haploxerert, respectively. After the first 2 weeks of measurement, the differences between the two soils reduced and the emission decreased until the end of the experimental period.

**Fig. 3:**
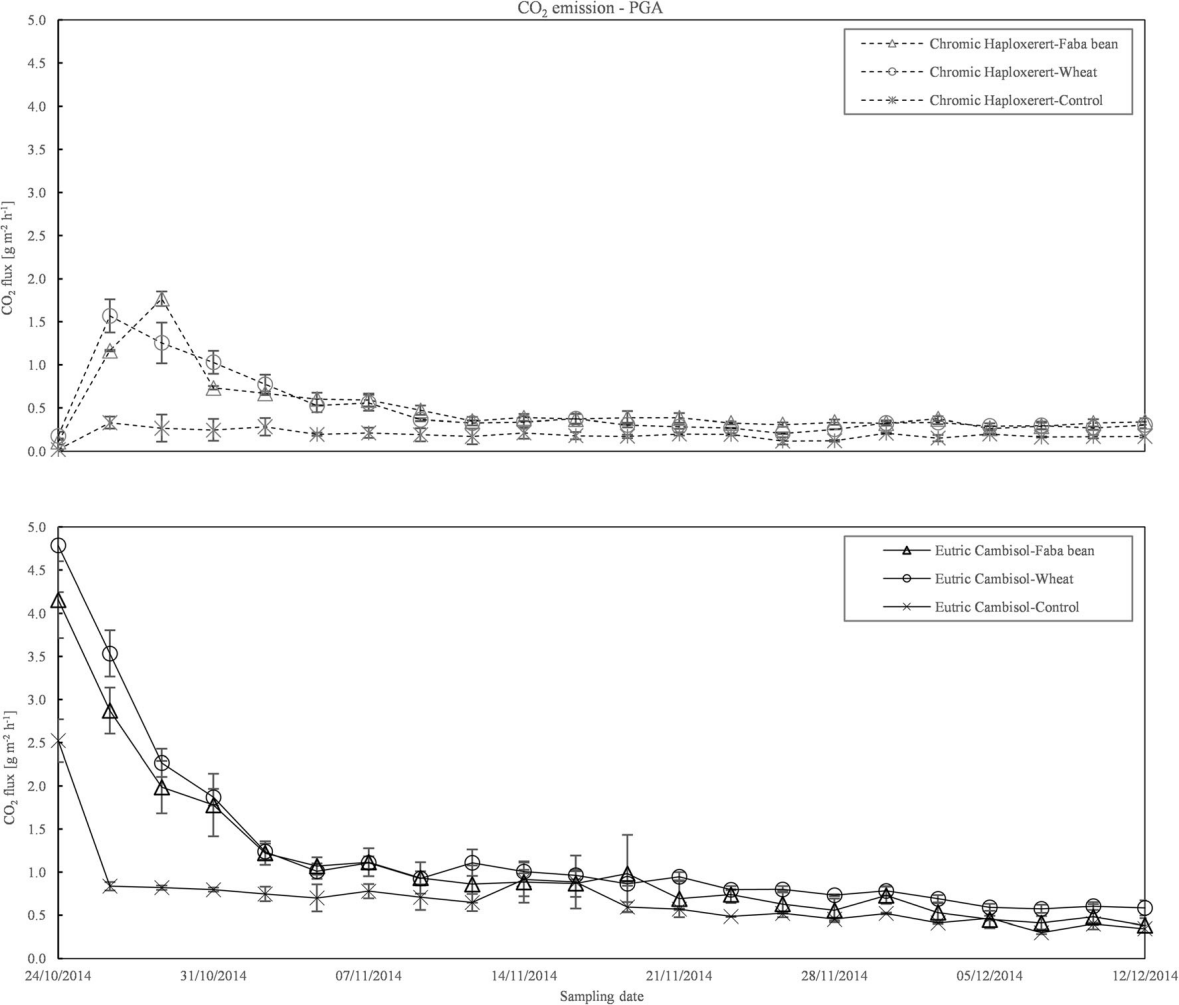
CO_2_ emission course from the Chromic Haploxerert and Eutric Cambisol soils amended with faba bean and wheat biomass, or unamended (control), measured with PGA during the experimental period. Data are means ± SE (*n* = 3)

The CO_2_ emissions measured with PGA showed a similar trend to those acquired by IRGA. However, in the first part of the experimental period, PGA emissions were slightly higher than those observed by the IRGA, especially from the Cambisol. In the second part of the experiment, no differences between the techniques were found (Fig. 3).

**Fig. 4:**
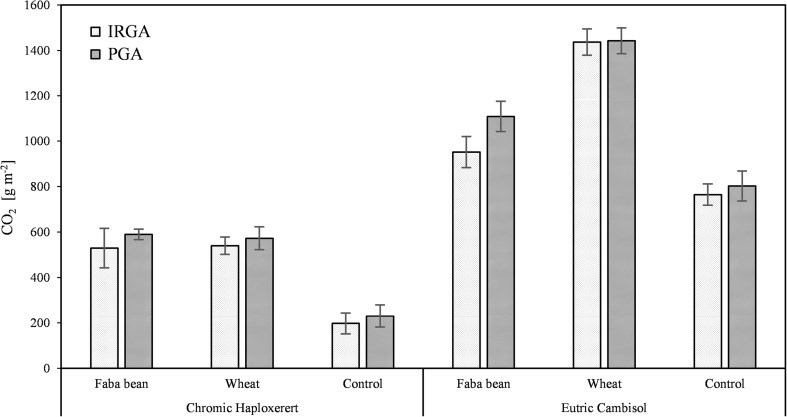
Total CO_2_ emission from the Chromic Haploxerert and Eutric Cambisol amended with faba bean and wheat biomass, or unamended (control), measured with IRGA and PGA. Data are means ± SE (*n* = 3)

Total CO_2_ emissions were 74% lower in the unamended Haploxerert (198 g CO_2_ m^−2^) compared to the Cambisol (765 g CO_2_ m^−2^). Addition of plant residues to the soil increased total emission to a different extent depending on the soil under study (interaction soil × residue type significant *p* < 0.001): in the Cambisol, addition of faba bean and wheat resulted in an increase of 24 and 88%, respectively, of the total CO_2_ emissions. In the Haploxerert, no differences were found between the kinds of biomass incorporated, which, on average, increased total CO_2_ emission by 171% compared to the unamended control (Fig. 4).

**Fig. 5:**
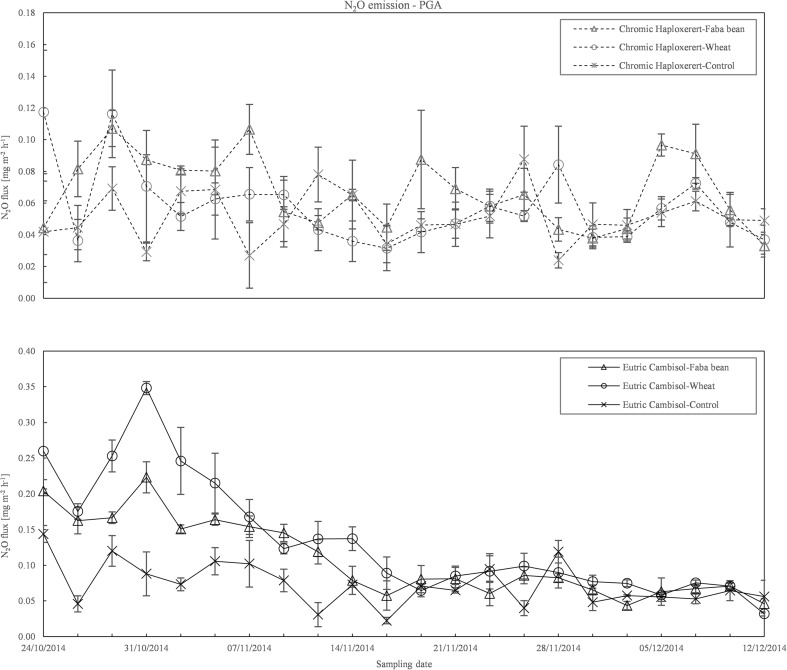
N_2_O emission course from the Chromic Haploxerert and Eutric Cambisol amended with faba bean and wheat biomass, or unamended (control), measured with PGA during the experimental period. Data are means ± SE (*n* = 3)

**Fig. 6:**
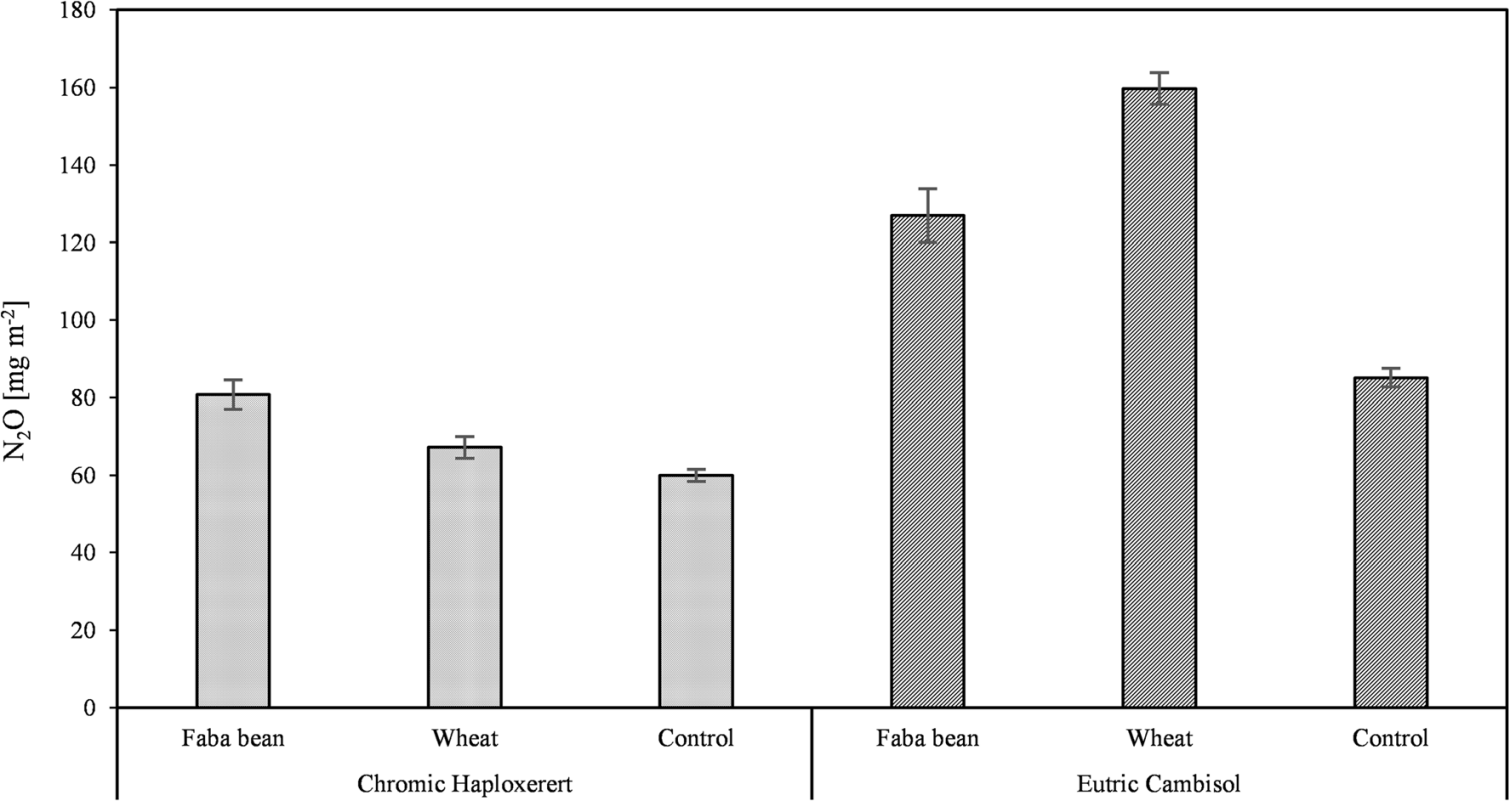
Total N_2_O emission from the Chromic Haploxerert and Eutric Cambisol soils amended with faba bean and wheat biomass, or unamended (control), measured with PGA. Data are means ± SE (*n* = 3)

Emissions of N_2_O during the experiment ranged from 0.022 to 0.348 mg m^−2^ h^−1^ (Fig. 5). However, there were large differences between soils with emissions of 0.024 to 0.117 mg m^−2^ h^−1^ and from 0.022 to 0.348 mg m^−2^ h^−1^ in the Haploxerert and Cambisol, respectively. The Cambisol reached a N_2_O emission peak at 7 days after the beginning of the experiment, whereas the Haploxerert soil showed a continuous and constant reduction of the N_2_O emission from the beginning of the experiment until the end of the trial. In addition, marked differences between amended and unamended soil were observed in Cambisol during the first half of the experiment. The highest fluxes were measured in both soils amended with wheat straw. Cumulative N_2_O emission in the unamended controls of the Cambisol soil was 30% higher than in Haploxerert soil (85.1 and 59.9 mg N_2_O m^−2^, respectively). Crop residue addition had a different effect in each soil (interaction soil × residue type significant *p* < 0.001). In the Cambisol, N_2_O emissions in the pots amended with wheat were 159.8 mg N_2_O m^−2^ (+88% more than the control) and that of the pots amended with faba bean was 127.0 mg N_2_O m^−2^ (+49% than the control). In the Haploxerert, faba bean-added pots emitted in total 80.8 mg N_2_O m^−2^ (+35% than the control) and that added with wheat 67.2 mg N_2_O m^−2^ (+12% than the control; Fig. 6).

### Comparisons of gas measurement techniques

Few differences were found for the IRGA and PGA in CO_2_ measurement when compared with that from the GC. The determination factor was 0.937 (*y*_GC_ = 1.0534*x*_IRGA_ − 0.0221 g CO_2_m^−2^ h^−1^) and 0.925 (*y*_GC_ = 0.9887*x*_PGA_ − 0.0095 g CO_2_m^−2^ h^−1^) for IRGA and PGA, respectively, and IoAd was 0.998 for both instruments.

With regard to the N_2_O measurement, the linear regression between GC and PGA showed a relatively high relationship between the results (*R* ^2^ = 0.90; (*y*_GC_ = 0.8993*x*_PGA_ − 0.0063 mg N_2_Om^−2^ h^−1^)), although PGA-N_2_O was, on average, 5.2% higher than the GC-N_2_O measurements. However, in this case, the IoAd was also 0.998.

The comparison between CO_2_ measurements obtained by IRGA and PGA across the entire experimental period (more than 600 measurements) showed a high correlation between the two instruments (*R* ^2^ = 0.95; IoAd = 0.996; (*y*_IRGA_ = 1.0118*x*_PGA_ − 0.0003 g CO_2_m^−2^ h^−1^). However, the cumulative CO_2_ emissions measured by PGA were on average 9% higher than those measured by IRGA. Differences in CO_2_ fluxes from the two soils were apparent from the different measurement techniques. Thus, although the overall CO_2_ fluxes measured by PGA were 6% higher than IRGA, such differences were up to 10% greater when the comparison was limited to the Haploxerert soil, and up to 17% when only the control plots were considered. In the Cambisol, the differences between the instruments were lower at around 5%.

## Discussion

### N_2_O and CO_2_ emission and soil properties

This study evaluated the effect of soil incorporation of two different plant residues on N_2_O and CO_2_ emissions. The characteristics of two soils were distinctly different, with the Cambisol having a low pH and high SOC, while the Haploxerert had a high pH and low SOC. Emissions and soil parameters varied according to both the kind of residue added and the soil type. The total CO_2_ and N_2_O emissions (measured by PGA), from the unamended Cambisol, were 249 and 40% higher than the unamended Haploxerert, respectively, suggesting large differences in biochemical and microbial activity between both soils driven by differences in soil physical and chemical properties. Moreover, the differences in CO_2_ emissions between the two soils followed the differences in stable C (TOC was 78% higher in the Cambisol than in Haploxerert) and readily available C (DOC in the Cambisol was double that in the Haploxerert). This latter form, although it may be preferentially utilized by soil microorganisms, can be protected by soil aggregates or adsorbed by mineral particles (Majumder and Kuzyakov 2010; Steinbeiss et al. 2008; Shi and Marschner 2014; Hadas et al. 2004). The higher CO_2_ emissions (per unit of carbon present in the soil) from Cambisol were nevertheless a reflection of differences in the carbon pools. Such differences suggest that the Haploxerert had a relatively low respiration rate, which may have been a consequence of protection by the higher clay content in the Haploxerert of SOC pools (Baldock and Skjemstad 2000; Krull et al. 2003; Lutzow et al. 2006; Alluvione et al. 2013; Six and Paustian 2014) and coupled with relatively low soil microbial activity due to a low free substrate availability. Another important aspect related to the clay content is its mineralogy; the Haploxerert is characterized by prominent swelling-shrinkage behaviour, which suggests that a high content of montmorillonite can slow down organic matter decomposition by absorption, interacting with soil microbes and their external enzyme activity or limiting oxygen diffusion (Vogel et al. 2015). In addition, a recent highly reliable model on SOC on the region, the Haploxerert in the present study, came from confirming that these kinds of soil (along with other Vertisols) have a high ability to stabilize the soil organic matter (Schillaci et al. 2017; Saia et al. 2017). CO_2_ and N_2_O fluxes reached a peak within the first week of incubation and were higher in the Cambisol than in the Haploxerert. The transient effects of the CO_2_ and N_2_O emission rates were likely to have resulted from increased gas diffusivity due to the soil disturbance in the establishment of the experiment and the rapid decomposition of the highly labile free organic fraction (either added or not) (Magid et al. 1999; Baggs et al. 2006). Crop residue distribution within the soil, as reported by several authors (Curtin et al. 1998; Jacinthe et al. 2002; Lian et al. 2016), stimulated and increased CO_2_ emissions but with different magnitudes in the two soils. In particular, the difference in CO_2_ emissions between soils was reduced when an organic residue (either faba bean or wheat) was added. The Cambisol emitted +88 and +152% more CO_2_ than the Haploxerert when faba bean and wheat residues were added, respectively. Similar differences were found for N_2_O emission between soils amended with organic residues. These findings are supported by research by An et al. (2015) where straw C input to the soil was more effective at stimulating microbial activity and extractable organic carbon in a low fertility soil, than in a high fertility soil, probably as a consequence of the starvation of the soil microbial community (Bastida et al. 2013) and also a possible effect of clay which increases the contact between the substrate and microorganisms. However, their experiment used a soil with a lower clay content (24.9%), and we expect that in the soil used in our study which was more rich in clay (52.5%), this effect was less important due to the absorption effects described earlier. Other studies have shown that an increasing clay content (achieved by making artificial soils) accelerated the decomposition rate of added organic matter supporting the concept that clay can have a primary role in influencing decomposition-stabilization processes in the soil regulating the nutrient availability for microorganisms, emissions and organic carbon stabilization and sequestration (Velthof et al. 2002; Six and Paustian 2014; Wei et al. 2014; Bajgai et al. 2014). Nitrous oxide emissions from the Haploxerert were affected also by soil clay content and its direct action on N immobilization processes, as observed also by Begum et al. (2014) in an experiment conducted in a same type of soil (Vertisol) with a comparable clay content (62%), closely linked to the stabilization of the organic matter and confirmed by the high NH_4_^+^-N/NO_3_^−^-N observed. Furthermore, as a result of the high cation exchangeable capacity of this soil (35 cmol kg^−1^), the addition of organic matter had no effect on the pH, whilst in the Cambisol, the wheat straw significantly reduced pH, most probably as a consequence of the nitrification process which may acidify soil due to the release of H^+^ ions (Van Miegroet and Cole 1984). This would have been promoted by the high degradability of wheat residues that produced a higher nitrate content in the soil and promoted gaseous emissions (both CO_2_ and N_2_O) compared to the soil where faba bean was added. In another experiment, Aye et al. (2016) using wheat and field pea, with a different C/N ratio, as residues in a soil with 29% clay found an increase in the decomposition process up to pH 7.4. However, in our experiment, although the pH of the Haploxerert was slightly higher (7.8), the lower DOC concentration and CO_2_ and N_2_O fluxes in Haploxerert suggest the lower decomposition rates that can be linked to the much higher clay content (52.5%) confirming the dominant influence of clay as a key factor in determining nutrient turnover and emissions in this soil. The original pH of the soil may have played a role in determining the magnitude of N_2_O emissions by the soil microbial community. As reported from Rousk et al. (2009), an acid pH at around 6 can stimulate fungal growth; fungi are recognized for not having the ability to synthesize nitrous oxide reductase and their denitrification end product is therefore N_2_O. Other studies have reported that fungi could contribute up to 18% of potential denitrification (Herold et al. 2012). Thus, pH differences may also have contributed to differences in N_2_O emissions from soils.

There was a clear correlation between CO_2_ and N_2_O emissions in both soils, although this was greater in the Cambisol, where oxygen depletion and CO_2_ emissions could have helped create anaerobic microsites in the soil increasing denitrification and N_2_O production (Gök and Ottow 1988; Aulakh et al. 1991; Begum et al. 2014; Nett et al. 2015). The mineralization rate of an organic residue added to the soil mostly depends on its C/N ratio and, to a lesser extent, to its lignin/N ratio and fibre content (Trinsoutrot et al. 2000; Nguyen and Marschner 2016; Cheng et al. 2015). However, in the present study, the difference in the C/N ratio of the residues used (38.6 in faba bean and 40.7 in wheat) does not explain the difference in soil mineral N concentration and CO_2_ and N_2_O emissions between the crop residues. Thus, it is more likely that mineralization rate of faba bean residues was lower than wheat residues due to the different lignin, acid detergent and NDF contents (+188, +66, +19%, respectively, in faba bean compared to wheat).

The incorporation of plant residues, either of wheat or faba bean, introduced contrasting effects on the NH_4_^+^-N and NO_3_^−^-N concentrations on each of the soils. The addition of plant residues increased the NH_4_^+^-N concentration of the Haploxerert, but not that of the Cambisol, and such an increase was more evident when wheat residues were added. At the same time, addition of plant residues reduced the total NO_3_^−^-N content of the Cambisol, but not that of the Haploxerert, and such an effect was more evident when faba bean residues were added. Such a result points to a net immobilization process in the soil due to consumption of N in order to decompose organic C (Corbeels et al. 2000; Jin et al. 2013). In the Haploxerert, a similar quantity of total CO_2_ was emitted after the addition of both crop residues, but the faba bean addition showed a slightly higher N_2_O emission than wheat addition treatment coupled with lower NO_3_^−^-N content at the end of the experiment. Thus, it is likely that in this soil, which was characterized by a lower soil microbial activity, the lower mineralization of faba bean residues led to a more constant availability of labile C and N, due stimulating bacterial and fungal activity along the experiment until the end and, as consequence, denitrification in soil microsites as reported from other authors (Deenik 2006; Shah et al. 2016). By contrast, wheat residues produced a rapid flush in emission in the initial phase of the experiment and have shown at the end of the experiment higher NH_4_^+^-N and NO_3_^−^-N concentration into the soil suggesting other limitations. This selective activity of microbes induced by the residue composition results in readily available straw C being used more rapidly while more recalcitrant and stable compounds are decomposed more slowly (Majumder and Kuzyakov 2010). In the Cambisol, both crop residues showed the same trend in gas emissions (CO_2_ and N_2_O), due to a direct effect of residue characteristics on decomposition and N availability. The rapid mineralization of wheat resulted lower DOC and higher NH_4_^+^-N and NO_3_^−^-N concentrations and a reduction in pH, as described earlier. In the case of faba bean, the higher presence of recalcitrant compounds, in particular lignin, slowed down nutrient release and decreased emissions.

### Comparison between gas flux measurement techniques

This study has clearly demonstrated that IRGA and PGA methodologies used to measure CO_2_ and N_2_O emissions provided data consistent with that measured by GC. The comparison of CO_2_ and N_2_O emission rates measured by IRGA and PGA was very strongly correlated with GC measurements, an observation also reported by other authors (Pumpanen et al. 2004; Iqbal et al. 2013; Nicoloso et al. 2013; Tirol-Padre et al. 2014). In particular, the same trend was observed for both gas fluxes measured in the Cambisol and Haploxerert, which were characterized by different patterns of CO_2_ and N_2_O emissions. Similar results to those observed in the present experiment were found for N_2_O fluxes by Iqbal et al. (2013), who reported slightly higher emissions with PGA than with GC (+5%), However, by contrast, we did not find any difference in CO_2_ flux measurements when comparing PGA and GC. Nicoloso et al. (2013) observed an overestimation of 18.6 and 13.6% compared PGA to GC, for CO_2_ and N_2_O, respectively; we did not find any differences between the techniques, which may have been due to the lower gas concentrations measured during our experiment. That also defined the positive effect of the compensation against water vapour and cross interference, the two main sources of interference on measurement, during the experiments.

With regard to the accuracy of CO_2_ emission data recorded by IRGA, if comparing our performance with those obtained from Pumpanen et al. (2004), the latter of which are based on CO_2_ concentration measurements, we obtained better results with very similar fluxes between IRGA and GC. The quality of data obtained from EGM-4 IRGA used in the present study was also confirmed by Mills et al. (2011), who found good similarity in soil respiration flux with a different IRGA type. However, PGA was found to have some limitations in reporting CO_2_ fluxes measured by IRGA in the first part of the experiment and monitoring the emissions of Haploxerert control in the later part of the experiment, showing some difficulty on measuring low and high peaks of emission producing a slight overestimation on data. At medium and low emission rates, the instrument performances were similar and this was also confirmed by GC. Taking into account the reliability of data, together with the speed of measurement and the capacity to obtain high-resolution temporal data, this study highlights the benefits of using online IRGA and PGA measurements in studies of residue decomposition. When applied in the field experiment, the short time required from IRGA and PGA to take a measurement of emission provides an opportunity to make more measurements permitting a higher spatial and temporal resolution. In the case of the PGA, the results produced had a considerable importance due to the possibility of this instrument to measure two or more gaseous compounds simultaneously (Horsley et al. 2014).

Finally, although the chamber techniques coupled with GC are considered the reference technique for the GHG monitoring, direct measurement by these devices eliminates many of the risks resulting from sampling pitfalls and sample storage that can negatively affect the measurements (Cowan et al. 2014; Tirol-Padre et al. 2014). For the specific application to GHG studies, the initial cost and maintenance can be lower than GC systems, requiring also less specialized staff to operate. The comparison of CO_2_ emissions rates measured by IRGA and PGA across the entire experimental period revealed, overall, that there were small differences between both methods.

## Conclusions

Soil plays a major role in controlling GHG emissions to the atmosphere and is a key determinant of emissions originating from plant residues. Our study demonstrated, when comparing two different soils, how specific properties, such as clay content and pH, can significantly alter decomposition, immobilization and gaseous emissions. These results have implications for developing low-C management practices, especially under organic farming systems where residue management could be a strategy to replace mineral fertilizers and limit C footprint. In Vertisols, which are widespread, but less well understood, CO_2_ and N_2_O emissions were strongly controlled by clay content limiting emissions, promoting C sequestration and N transfer to next crop cycle. Although many studies on the decomposition of residues have focused on C/N ratios, this study highlights the importance of fibre compounds, often referred to as secondary, on determining soil CO_2_ and N_2_O emissions and as their effect can change in relation to the soil characteristics. In particular, in soil with high organic carbon contents and microbial activity such as a Cambisol, the crop residue type determined the total emission. There was a unique trend for higher emissions of both gases (CO_2_ and N_2_O) in the presence of more decomposable wheat than with recalcitrant faba bean. In Haploxerert, by contrast, the slower decomposition of crop residues resulted in a similar CO_2_ release from the different residues, but slightly higher N_2_O emissions from faba bean.

The direct comparison between IRGA and PGA and their validation with GC confirmed that these two techniques are equivalent in providing reliable data for long-term monitoring, and this occurred under various conditions (differing soil type residue addition). This result is important when considering that GC-based methodologies need a number of sample steps from gas collection, transport, sample storage and analysis, each of which can potentially add error to the measurement. In addition, GC-based methodologies are not able to provide a continuous measurement of the GHG emissions and thus are poor at quantifying temporal variability. By contrast, the high sensitivity of IRGA and PGA, range and ease of application and number of gases analysed (including water vapour) allow a better monitoring of the radiative force of the soil while eliminating many of the risks of the GC-based methodologies.
